# Infectivity versus Seeding in Neurodegenerative Diseases Sharing a Prion-Like Mechanism

**DOI:** 10.1155/2013/583498

**Published:** 2013-09-25

**Authors:** Natalia Fernández-Borges, Hasier Eraña, Saioa R. Elezgarai, Chafik Harrathi, Mayela Gayosso, Joaquín Castilla

**Affiliations:** ^1^CIC bioGUNE, Parque Tecnológico de Bizkaia, Derio, 48160 Bizkaia, Spain; ^2^IKERBASQUE, Basque Foundation for Science, Bilbao, 48011 Bizkaia, Spain

## Abstract

Prions are considered the best example to prove that the biological information can be transferred protein to protein through a conformational change. The term “prion-like” is used to describe molecular mechanisms that share similarities with the mammalian prion protein self-perpetuating aggregation and spreading characteristics. Since prions are presumably composed only of protein and are infectious, the more similar the mechanisms that occur in the different neurodegenerative diseases, the more these processes will resemble an infection. *In vitro* and *in vivo* experiments carried out during the last decade in different neurodegenerative disorders such as Alzheimer's disease (AD), Parkinson's diseases (PD), and amyotrophic lateral sclerosis (ALS) have shown a convergence toward a unique mechanism of misfolded protein propagation. In spite of the term “infection” that could be used to explain the mechanism governing the diversity of the pathological processes, other concepts as “seeding” or “*de novo* induction” are being used to describe the *in vivo* propagation and transmissibility of misfolded proteins. The current studies are demanding an extended definition of “disease-causing agents” to include those already accepted as well as other misfolded proteins. In this new scenario, “seeding” would be a type of mechanism by which an infectious agent can be transmitted but should not be used to define a whole “infection” process.

## 1. Introduction

It has been some time since the old axiom, “one protein—one structure,” became obsolete [1]. This is most obvious for prion scientists that try to understand how different protein structures can originate from the same primary sequence. The idea that proteins must acquire a specific and unique conformation has shifted due to biochemical and structural evidence from neurodegenerative diseases showing that different folding states of the same protein are essential in certain biological processes. This concept was nicely expressed by Batch and colleagues: “The misfolding and aggregation of proteins is often an accident waiting to happen. Consequently, organisms have developed sophisticated chaperone and quality-control systems to limit abnormal protein interactions and the accumulation of toxic aggregates” [2]. The ability of proteins to reach different isoforms has crucial consequences in the cell and in the whole organism as demonstrated in prion diseases, neurodegenerative pathologies where the prion protein (PrP) misfolding process is the key event.

We will review the behavior of different proteins implicated in several neurodegenerative diseases selecting those with a high impact on our society and comparing them to prion diseases as reference. The term “prion-like” will be used to describe molecular mechanisms that share similarities with the mammalian prion protein self-perpetuating aggregation and spreading characteristics. Since prions are presumably composed only of protein and are infectious, the more similar the mechanisms that occur in the different reviewed diseases are, the more these processes will resemble an infection.

## 2. *In Vitro* Propagation

Essentially, any protein could show a prion-like propagation if it were able to acquire a characteristic folding that could be induced to other surrounding proteins with identical or similar amino acid sequences. Thus, the biological information would be transferred protein to protein through a conformational change. Although PrP in transmissible spongiform encephalopathies (TSEs) is the most representative protein of this phenomenon, APP (amyloid precursor protein) and tau in Alzheimer's disease (AD) as well as in frontotemporal dementias (FTD), *α*-Synuclein in parkinson's diseases (PD) and superoxide dismutase 1 (SOD-1), and the 43KDa TAR (trans-activator regulatory) DNA binding protein (TDP43) in amyotrophic lateral sclerosis (ALS) are also involved in protein misfolding propagation. All these proteins are implicated in neurodegenerative diseases in which the pathogenic process had been associated with the presence of amyloid deposits predominantly composed by misfolded proteins [3–6]. Although these diseases share a neurodegenerative process that leads to death, the type of proteins involved, their localization and the manner in which they accumulate in amyloid fibers are not shared.

*In vitro* studies were and still are of vital importance for understanding the mechanisms governing misfolding propagation from one protein to another. The process by which a protein changes from its initial folding to a structure prone to amyloid formation was initially described by Jarrett and Lansbury. The so called “seeding,” first observed in PrP conversion to amyloid fibrils, proved to be similar to the “one-dimensional crystallization” mechanism, a nucleation-dependent phenomenon that is common to other amyloid forming proteins such as *β*-amyloid (A*β*) [7]. The same *in vitro* studies also showed that propagation implies protein aggregation and that in some cases the aggregation that follows misfolding can occur spontaneously. Jarrett and Lansbury described the key parameters that characterize the dynamics of aggregation such as the lag phase and the aggregation rate [8]. Although their experiments were focused on PrP and *β*-amyloid, we now know that those parameters can be used in all the proteins mentioned above and that they all share the ability to propagate misfolding *in vitro*.

One of the first lines of evidence that showed a similar *in vitro* behavior for the prion protein and the *β*-amyloid peptide was provided by Come and collaborators. They pointed out that the *β*-amyloid peptide contains a C-ter region similar to PrP(96-111), which is necessary for amyloid formation and possibly responsible for protein aggregation initiation *in vivo*. These proteins showed in *in vitro* fibrillization experiments the existence of a kinetic barrier to amyloid formation expressed as a lag phase analogous to crystal growth. These results suggested the formation of an ordered nucleus, which is a rate-determining step for aggregation and is followed by rapid fibril growth. Seeding was also demonstrated by incubation with preformed fibrils, which shortened considerably the lag phase and is consistent with a nucleation-dependent mechanism [7, 8]. Other studies have confirmed the seeding phenomenon described previously for A*β* and tau [9–14] and well characterized by Stöhr and collaborators for PrP, showing that seed-enhanced growth could be achieved in homogeneous solution and could be enhanced by sonication. They proposed a mechanistic model of fibrillization that included the presence of several intermediate structures [15]. In a similar manner, a nucleation-dependent mechanism for *α*-synuclein fibrillogenesis was also described. As the previous one, it consists of an initial lag phase (nucleation) followed by a growth phase (elongation) and a constant state phase where the organized aggregate and the monomer are at an equilibrium [16]. The nucleation-dependent process may be the rate-limiting step in PD during the generation of Lewy body (LB) *α*-synuclein fibrils. Serpell and collaborators also showed the conformational change of *α*-synuclein from an *α*-helix structure to a *β*-sheet conformation during assembly *in vitro*. In this study, different types of recombinant *α*-synuclein were used: carboxy-terminally truncated human *α*-synuclein (1–87) and (1–120), wild-type human *α*-synuclein and the A53T mutant human *α*-synuclein. Surprisingly, wild-type and A30P mutant human *α*-synucleins showed slower rates of aggregation than the truncated proteins. The fibrils generated as a result of shaking were identical to the fibrils extracted from dementia with Lewy bodies and multiple system atrophy brains [17]. *α*-synuclein fibril formation, previously characterized by Spillantini and coworkers from the substantia nigra of idiopathic PD patients, had been reproduced *in vitro*. The labeled extracted structures corresponded principally to single filaments; however, small clusters of filaments were also observed. Fibrils showed a variable morphology: straight and unbranched with diverse length and width [6].

Interestingly, prions occur in the form of different strains that show distinct biological and physicochemical properties, even though they are encoded by prion proteins with the same amino acid sequence, albeit in presumably different conformations. Recent studies focused on polymerization/fibrillization of *β*-amyloid and tau also demonstrated the existence of structurally different aggregates visualized by electron microscopy (EM) and atomic force microscopy (AFM). These protein aggregates also behaved differently in cell culture [11–13, 18]. These studies can be considered pioneers identifying the strain phenomenon in AD and other tauopathies. Similarly, Wang and coworkers showed that tau fragments prepared by endogenous proteases aggregated spontaneously *in vitro* and propagated to tau fragments as well as to full-length tau in a similar way to the one described in prion propagation [19, 20].

A common feature in the neurodegenerative diseases is the existence of mutations in the proteins favoring (with some exceptions) protein misfolding. This *in vivo* event is responsible for the genetic and familial forms of several diseases and usually gives rise to a spontaneous early onset [21–26]. Many studies have proved that this occurrence can also be mimicked *in vitro*. Mutations in APP (occurring in early AD) alter mostly the processing of this precursor protein by secretases, leading to the release of greater amounts of A*β* peptide or the alteration in the ratio of A*β* types. Jarret and collaborators tested the *in vitro* aggregation kinetics of some of the most abundant variants of A*β* found in senile plaques showing distinct rate of amyloid formation from *in vivo* [27]. Around 10% of tauopathies are familial forms of FTD and are associated with the presence of mutations. The wild-type and the most characteristic mutant forms of tau were studied by Frost and coworkers *in vitro*. Fibrils composed of mutant or wild-type tau showed different structures by FTIR (fourier transform Infrared spectroscopy). Seeding of wild-type tau with mutant fibrils led to a new structure different from that formed with wild-type tau seed, which can explain the phenotypic diversity of tauopathies [9]. A similar observation was described by Narhi and coworkers in Parkinson's disease-related studies. Despite that both wild-type and mutated *α*-synucleins can be used to *in vitro* assemble fibrillar aggregates with a cross-*β*-sheet conformation, aggregate formation is accelerated when mutated *α*-synuclein (characterized in PD patients) is used [22]. For instance, fibrils composed of *α*-synuclein A30P mutant acting as seed accelerate the nucleation-dependent fibrillization of the wild-type protein perpetuating the “A30P strain” properties generated *in vitro* [28]. Likewise, ALS-associated mutations that promote *in vivo* toxicity also accelerate *in vitro *aggregation of highly purified TDP-43. This protein, a pathological hallmark of ALS, is considered as inherently aggregation prone [29].

Although the tertiary and quaternary protein structures involved in aggregate formation among the diversity of neurodegenerative pathologies are likely different, the antibody recognition of a common aggregated structure suggests a similar oligomeric organization [30]. This fact is extremely interesting since it allows future studies on therapeutic approaches and predicts common propagation mechanisms of these diseases.

## 3. Cell-to-Cell Propagation

While* in vitro* misfolding protein propagation is not affected by protein localization or by the existence of cellular components/factors, it might be possible that *in vivo* propagation is impeded at the cellular level. In order to study the “prion-like” phenomenon that implies the spreading of the self-perpetuating protein aggregates also from cell to cell, a diversity of studies have been performed using cell cultures. Thus, misfolded tau protein was propagated cell to cell after seeding by pathological tau conformers leading to pathogenesis of Alzheimer-like tangles in cells [14, 18]. *In vivo*, cell-to-cell propagation was unequivocally demonstrated using a tau transgenic model in which overexpression of human tau P301L was restricted to the entorhinal cortex (EC-II) area. Tau proteins spread from neuron to neuron into different brain areas coaggregating with mouse endogenous tau, in a way similar to prions [31]. A recent study has demonstrated that soluble oligomeric A*β* can also be transmitted neuron to neuron depending on direct neuritic connections, following a prion-like intercellular spread. The authors of this study propose macroautophagy as a potential mechanism for disease spreading, similar to endolysosomal and lysosomal exocytoses described for prions [32].

There are also several lines of evidence that suggest *α*-synuclein as a candidate to be the pathogenic factor implicated in the prion-like spread of PD pathology. Freundt and coworkers showed neuron-to-neuron transmission of *α*-synuclein fibrils through axonal transport [33]. Fibrillar *α*-synuclein internalization in primary neurons followed by the transport of these fibrils to the cell bodies of second-order neurons was observed. Moreover, exogenous *α*-synuclein fibrils were an efficient seed for the formation of Lewy-body-like intracellular inclusions in cultured cells [34]. *In vitro* preformed fibrils were added into *α*-synuclein overexpressing cells and the formation of insoluble intracellular inclusions was evaluated. Aggregates very similar to PD Lewy bodies composed principally of *β*-sheet, hyperphosphorylated and polyubiquitinated *α*-synuclein were observed in subcellular localization [34].

The prion-like behavior in ALS was studied using cell cultures expressing ALS-causing mutant SOD-1 or TDP-43. The cells were seeded using *in vitro* preformed SOD-1 aggregates that penetrated through macropinocytosis. As a consequence, the pathological misfolding of the endogenous soluble mutant protein was triggered. Aggregates were effectively transferred to adjacent cells via exosomes or nanotubules and continued growing even after the misfolded proteins acting as seed were eliminated. This suggests a cyclical self-perpetuating behavior, mimicking what happens in prion diseases [35–37].

## 4. When the *In Vivo* Propagation Can Also Be Transmissible

The transmission ability from an individual to another is one of the main hallmarks of prion diseases. Transmissibility requires *in vivo* propagation, which should occur somewhere in the cell. Although the most probable place is the cytoplasmic membrane where GPI-anchored PrP is located, it is not restricted there because *in vivo* propagation has also been observed in models that express GPI-less secreted PrP in which misfolding and accumulation occur in the extracellular matrix [38]. Thus, before discussing the transmission capacity of these diseases, we should focus on their ability to spread throughout the central nervous system. In AD patients, one of the first changes observed in the brain is the deposition of A*β* plaques. Therefore, their apparition and spreading have been widely studied. One of the most detailed studies analyzed 83 brains from healthy as well as AD affected donors in different disease stages to correlate depositions with disease progression [39]. A*β* depositions initially appeared in basal portions of the isocortex, but as the disease progressed, the whole isocortical area became affected with a mild presence of plaques in the hippocampus, finally spreading to subcortical areas. The neurofibrillary tangles (NFT) and neuropil threads (NT) formed by tau resulted in much better indicators of disease progression, with a characteristic distribution pattern with much less variation among individuals than A*β* plaques. The latter were initially confined to a single layer of the transentorhinal region, followed by a severe involvement of the entorhinal and transentorhinal and finally arriving to isocortical destruction [39]. The degree of disease development could be measured through the amyloid spreading, being this behavior of deposit propagation characteristic also in some TSEs.

More recently, Seeley and collaborators used functional neural-network sensitive neuroimaging methods to analyze the atrophy patterns of those networks in brains of donors with 5 different dementias, including AD and FTPD. They demonstrated that as in the case of TSEs, where there are many lines of evidence of direct propagation of deposits along transsynaptic connections [40], other neurodegenerative diseases are also related to neural-network dysfunction. Each dementia syndrome would have its constant pattern of affected regions that would match different neural networks. This suggests that many disease-related proteins such as *β*-amyloid, tau, or *α*-synuclein are able to misfold, aggregate, and spread with specific brain networks [41].

A similar *in vivo* propagation was also proposed in Parkinson's disease. The disease may initiate in the periphery/enteric nervous system, accessing the central nervous system (CNS) through retrograde transport along neuronal projections from the gastrointestinal tract. After the Lewy pathology is transferred to CNS, it ascends from the lower brainstem through susceptible regions of the midbrain including the substantia nigra and the forebrain and finally spreads to the cerebral cortex at later stages of the disease. Alternatively, pathology may begin at the anterior olfactory structures spreading to midbrain and cerebral cortex. As the pathology progresses, the severity of the lesions in the susceptible regions and the clinical manifestations increase [42, 43]. 

Although *in vivo* propagation through the spinal motor neurons of the ALS-related misfolded proteins (SOD-1 and TDP-43) has been hypothesized, an *in vivo *prion-like propagation has not been demonstrated yet [37].

Despite the spreading mechanisms demonstrated by some of the mentioned misfolded proteins that resemble a prion-like propagation, their “labelling” as transmissible or infectious proteins would require verifying if this phenomenon can also be stimulated by their exogenous inoculation. As a result, pathological processes that would occur spontaneously could be accelerated or, in other cases, a disease that would not happen naturally could be initiated* de novo*.

Each of the neurodegenerative diseases described in this review is being studied over several animal models. Most of them are based on transgenic mouse models that overexpress the human disease-causing proteins. Overexpressed proteins carrying one or more mutations recreate the human disease with shorter progression times. The majority of those models reproduce the spreading of protein misfolding differing in propagation rates, affected areas, and the number or type of implicated proteins. Thus, they are excellent tools to study transmissibility of exogenous aggregates and to verify prion-like behavior based on the ability of propagation and self-perpetuation of the disease-related proteins.

One of the first many lines of evidence of A*β* transmissibility was described by Kane and coworkers in 2000. In this study, *β*-amyloid precursor protein transgenic mice (Tg2576) were intracerebrally infused with diluted supernatants of autopsy-derived neocortical homogenates from Alzheimer's patients. While Tg2576 mice develop *β*-amyloid deposits spontaneously at 9 months of age [44], the inoculated mice showed a significant reduction between 3 and 8 months in the *β*-amyloid plaque's onset. Wild-type or Tg2576 mice infused with healthy human brain did not show any A*β* deposition in the brain. As Tg2576 spontaneously favor this phenomenon it was considered as “seeding,” far from the idea of “infection” designated for prions after inoculation [45]. 

Few years later different Alzheimer transgenic mouse models were also inoculated with autopsied Alzheimer's patient brain extracts. These studies show that *β*-amyloidogenesis is highly dependent on the expression of human APP of the different transgenic mouse models (the host) and on A*β* status (the agent). It was suggested that the variable seeding efficacy of these *in vivo* studies compared to *in vitro* studies was due to the occurrence of various A*β* conformations with partially distinct biological activities, comparable to prions [46].

In an attempt to recapitulate other prion features, the proteinase K resistance of A*β* extracts prepared from aged transgenic mouse brains was compared to that of synthetic fibrillar A*β*. As it has been described in TSE, a higher resistance of the brain-derived A*β* was observed. Surprisingly, PK digested derived A*β* retains the ability to induce *β*-amyloid deposition in APP23tg mice similar to what happens in prions [47].

The previous transmission studies were based on transgenic mouse models that, within the required time, spontaneously develop the same processes that are trying to be induced. This suggests that the newly formed *β*-amyloid depositions could be interpreted as an acceleration of the amyloidogenesis rather than a *de novo* induction. In order to address this, Morales and collaborators carried out new transmissibility experiments in which wild-type human APP overexpressing transgenic mice that do not develop spontaneous amyloidogenesis were inoculated with human Alzheimer's disease brain extracts. After long incubation times, the animals showed *de novo β*-amyloid depositions induced by contact with A*β* extracts, ruling out a seeding acceleration and suggesting an infectious prion-like propagation [48]. The use of this model has been very useful to demonstrate that the misfolded proteins can be *de novo* induced exogenously and refute the idea that the event can be explained as a simple seeding phenomenon. This is one of the first powerful lines of evidence demonstrating “infectivity” versus “seeding” mechanism. However, those animals overexpress the wt human APP and it could be argued that the seed was shortening a lag phase (likely longer than the animal lifespan) in the “primed” mice by overexpression.

The most efficient infection route for prion transmission is the intracerebral inoculation. Other parenteral inoculations (intravenous, intraperitoneal, etc.) although less efficient can also be used for prion transmission. Although previous successful A*β* inoculations were performed by direct intracerebral infusion of different preparations of *β*-amyloid, other peripheral routes were also used [49]. These results remind of those observed using some prion strains which, despite an efficient intracerebral transmission, fail on the intraperitoneal and/or oral route [50]. Recent peripheral inoculation experiments have shown for the first time that *β*-amyloid can be transmitted intraperitoneally when enriched *β*-amyloid extracts are used. This fact recapitulates prion transmission studies where barriers can be overcome by improving mouse models, enriching inocula, extending expected incubation times, or increasing the number of animal passages [51].

While most of the animal models used to study A*β* propagation were based on transgenic mice, a rat model has also been used to evaluate the pathological effect of exogenous A*β* extracts intracerebrally inoculated. This model as well as the one used by Morales and colleagues is characterized by the absence of spontaneous A*β* deposition. Both studies concluded that *β*-amyloid deposits can be generated *de novo* as in a genuine prion infection [48, 52].

Another very interesting model is the marmoset, a New World monkey that naturally develops A*β* plaques when aged. A 20-year experiment confirmed that misfolding protein deposits onset can be reduced following intracerebral inoculation of exogenous *β*-amyloid [53].

The prion-like propagation phenomenon is not limited to A*β* peptide in AD. Tau protein follows similar mechanisms of fibrillization both in AD and in FTPD and other tauopathies. Prion-like *in vivo* seeding and spreading of tauopathies has been demonstrated using two different transgenic mouse lines, ALZ17 and P301S. While the first transgenic line expresses human wild-type tau and does not develop spontaneous tau aggregates, the second one expresses human P301S mutant tau (linked to a familial form of FTPD) and shows abundant tau inclusions. When ALZ17 mice were inoculated intracerebrally with brain extracts from aged P301S animals, assembly of wild-type human tau into filaments was observed, as well as spreading of pathology from the site of injection to adjacent brain regions [54]. In a similar way, Sydow and colleagues showed that tau pathology can be triggered in an inducible transgenic mouse model expressing aggregation prone human tau mutant (containing a ΔK280 mutation, associated with FTDP that aggregates rapidly *in vitro*) along with endogenous mouse tau. This model developed mixture tangles composed of both human and murine tau rapidly after induction of human tau mutant. The mixture of tau species turned richer in mouse tau when the expression of human tau was switched off. The interspecies coaggregation ability of tau reminds of another well described characteristic of prions [10].

Interactions between both A*β* and tau observed in transgenic models support the amyloid cascade hypothesis of AD and suggest that polymerized A*β* forms trigger a cascade of events leading to the formation of tau NFT. Transgenic mouse models expressing human tau mutant P301L, which is aggregation prone, were inoculated with A*β*-containing brain extracts purified from aged APP23 mice. A strong tau deposition even in regions far from injection site was induced. When both transgenic mouse models were crossbred, an induction of early tau deposition greater than the one induced in older simple tau transgenic mice was observed. However, the A*β* depositions were similar to those observed in the simple APP23 transgenic mice, confirming strong parallelism with prion diseases [55].

The first evidence of the similarity between Parkinson's and prion diseases had a different origin compared to the transmission studies conducted in Alzheimer's disease. Nevertheless, it was unquestioned to ascribe to *α*-synuclein a prion-like behavior in terms of its ability to be propagated *in vivo* and to be transmitted among individuals. Fourteen years after transplantation of human fetal neurons in a PD patient it showed postmortem pathological changes typical of PD in the grafted neurons located in the putamen. Numerous grafted nigral neurons showed aggregated Lewy-body-like structures with *α*-synuclein and ubiquitin [56]. This study was the first evidence that the propagation of a misfolded protein in PD could be explained through a prion-like mechanism. Similar studies confirmed that transplanted dopaminergic neurons developed PD pathologic changes as a consequence of their proximity to the already affected neurons from the PD patient [57, 58]. Grafted cells contained posttranslationally modified and aggregated *α*-synuclein suggesting that aggregation and deposition in transplanted dopaminergic neurons were caused by the misfolded *α*-synuclein in the host brain, which was transmitted to grafted cells [58]. In all these studies the “seeding” process was carried out in an opposite direction than previously shown in the transmission experiments of AD. The remaining misfolded *α*-synuclein in PD patients was able to self-propagate using fresh *α*-synuclein from grafted naïve tissues.

The transplantation studies done in human were nicely replicated in different mouse models. Thus, the injection of mouse cortical neuronal stem cells into the hippocampus of transgenic mice overexpressing human *α*-synuclein triggered the direct transmission of *α*-synuclein from host to grafted cells as was previously described in the prion field [59, 60]. An *in vitro* coculture model was used to demonstrate that *α*-synuclein was transmitted via endocytosis to neighboring neurons forming LB-like juxtanuclear inclusions. A failure of the protein quality-control systems, especially lysosomes, promoted the accumulation of transmitted *α*-synuclein and related to inclusion formation demonstrating cell-to-cell transmission of *α*-synuclein aggregates [61].

More recently, two different models showed that extracellular *α*-synuclein was taken up by cells through endocytosis and interacted with intracellular *α*-synuclein. The first model was created using a viral vector to engineer rat nigral neurons to overexpress human *α*-synuclein that subsequently was transported to the striatum. Rat ventral mesencephalic neurons were grafted into the striatum of these mice showing a frequent transfer of *α*-synuclein from the rat brain to grafted dopaminergic neurons [62]. The second model was based on the use of grafted wild-type mouse embryonic mesencephalic neurons in the striatum of mice overexpressing human *α*-synuclein. Six months after grafting, the presence of intracellular human *α*-synuclein immunoreactive punctae was observed in few grafted cells [63].

In a similar way to previous transmission studies using prions or AD as a source of “propagative agent,” young TgM83 mice (transgenic mouse of synucleinopathy expressing human A53T mutated *α*-synuclein) were inoculated intracerebrally with brain homogenates from older TgM83 mice affected by the synucleinopathy. Prion-like acceleration of *α*-synucleinopathy was observed through the presence of both *α*-synucleins hyperphosphorylated and aggregated, together with a decrease on the survival time of mice. By contrast, there was no evidence of *α*-synucleinopathy in *α*-synuclein knockout mice suggesting an important role for *α*-synuclein protein in the transmission of pathology from affected to unaffected areas, as what happens with PrP during prion propagation [64]. A similar experiment using the same model is described later showing again that the intracerebral inoculation of pathological *α*-synuclein initiates a rapidly progressive neurodegenerative disease. Animals were injected with brain homogenates of aged symptomatic animals showing abundant LB-like *α*-synuclein pathology into the neocortex and striatum of young healthy animals. Abundant *α*-synuclein lesions were widespread throughout the CNS 90 days after injection. In the same study, *α*-synuclein preformed fibrils (PFFs) previously generated *in vitro* from human wild-type full-length *α*-synuclein were also inoculated. *In vitro* PFFs were also able to initiate and propagate *α*-synuclein pathology in the same manner as TgM83 sick mice [65].

The last pieces of evidence showing a prion-like behavior in PD were obtained using wild-type mice. Animals were injected in the dorsal striatum with synthetic murine *α*-synuclein fibrils initiating a cell-to-cell transmission of pathologic *α*-synuclein and PD-like Lewy pathology in anatomically interconnected regions. This LB accumulation produced a progressive loss of dopamine neurons in the substantia nigra reducing dopamine levels and therefore generating a progressive performance deterioration of impaired motor coordination and balance [66]. The efficient PD-like pathology transmission was unlikely due to the use of murine fibrils in a wild-type model since the human preformed *α*-synuclein fibrils and sarkosyl-insoluble *α*-synuclein purified from PD patient brains also efficiently induced LB-like pathology with abnormal phosphorylated *α*-synuclein-positive structures. Nevertheless, the percentage of mice developing PD pathology was lower using human *α*-synuclein than using mouse *α*-synuclein as an inoculum, evidencing a prion-like species barrier phenomenon [67].

Triggering a disease in wild-type animals after the exogenous inoculation of any kind of “agent” should be considered the definitive probe of “agent” transmission independently of the mechanism by which the animals develop the disease.

There is still no evidence in animal models that would make certain that the ALS-related proteins (SOD-1 and TDP-43) can be propagated or transmitted *in vivo* following a prion-like mechanism.

## 5. Why Talk about “Seeding” or “*De Novo* Induction” When It Is Being Described as an Infection?

During the last decade, research groups working on different neurodegenerative disorders have carried out *in vitro* and *in vivo* experiments that have been previously performed to study prion diseases. As a consequence, a convergence among this type of diseases toward a unique mechanism of misfolded protein propagation have been observed. This fact is triggering the use of new common therapeutic approaches (bexarotene, Anle138b, etc.) or common diagnostics tools [30, 68, 69].

Despite the data already accumulated, there are crucial studies that should be implemented or performed in a more prion-like style. Strain and species barrier phenomena are still two important prion features on which the next studies in neurodegenerative disorders should be focused for a complete recapitulation of prion diseases.

It is surprising that among all the reviewed articles discussing prion-like mechanisms, none of them mention “infectivity” as a mechanism to understand the diversity of the pathological processes. On the other hand, both “seeding” and “*de novo* induction” are concepts frequently mentioned to describe the *in vivo* propagation and transmissibility of misfolded proteins, an identical *in vivo* process that in the prion field is considered “infection”. There are several reasons why this might be explained. (i) It took more than 30 years for most of the prion community to agree with the “protein only hypothesis” [70]. (ii) Although “infectious” is not synonymous of “contagious,” both terms could be considered equal in certain contexts. Thus, the assumption that common neurodegenerative diseases should be considered as infectious diseases could be perceived as unnecessarily frightening to the population, and (iii) the lack of a wider definition for “infectivity” able to encase all the disease-causing agents.

Far from conventionalisms and if we assume that Koch's postulates must be adapted to accommodate etiologically atypical diseases [71], we should extend the definition of “infectious agent” to include parasites, bacteria, virus and viroids, prions, and, why not, other misfolded proteins. Thus, we would suggest the following definition: “Infection is a process by which a self-propagating agent that exogenously penetrates or is generated spontaneously causes disease or damage as a consequence of its intrinsic capacity to make identical or similar copies of itself through a diversity of mechanisms requiring or not exogenous components.” According to this and trying to answer the question opened from the title of this review, “seeding” would be a type of mechanism by which an infectious agent can be transmitted but should not be used to define a whole “infection” process.
